# Characteristics and Benefit Design of Veteran Medicare Advantage Affinity Plans

**DOI:** 10.1001/jamahealthforum.2025.0159

**Published:** 2025-03-28

**Authors:** Allison Dorneo, Yanlei Ma, Melissa M. Garrido, Steven D. Pizer, Paul R. Shafer, Thomas C. Tsai, Austin B. Frakt, Jose F. Figueroa

**Affiliations:** 1 Department of Health Law, Policy and Management, Boston University School of Public Health, Boston, Massachusetts; 2 Boston VA Healthcare System, Boston, Massachusetts; 3 Department of Health Policy and Management, Harvard T. H. Chan School of Public Health, Boston, Massachusetts; 4 Department of Surgery, Brigham and Women’s Hospital, Boston, Massachusetts; 5 Department of Medicine, Brigham and Women’s Hospital, Boston, Massachusetts

## Abstract

**Importance:**

Recently, there has been an emergence of veteran Medicare Advantage affinity plans (VMAPs) marketing to veterans, including those dually covered by the Veterans Health Administration (VHA). To date, limited evidence exists characterizing what benefits VMAPs offer and their veteran enrollees.

**Objective:**

To examine plan-level differences between VMAPs and other Medicare Advantage (MA) plans and characteristics of their veteran enrollees.

**Design, Setting, and Participants:**

This cross-sectional study compared the plan benefit design, supplemental benefit offerings, and veteran enrollee characteristics of all VMAP and other MA plan enrollees in 2022 using standardized mean differences (SMDs). VMAPs were identified based on military-associated words in their plan name and further validated through a web-based search. Data were analyzed from April 2023 to August 2024.

**Exposure:**

VMAP designation.

**Main Outcomes and Measures:**

Plan-level characteristics, supplemental benefits, and veteran enrollee characteristics.

**Results:**

The sample included 188 VMAPs with 179 449 veteran enrollees and 3442 other MA plans with 954 581 veteran enrollees. A total of 1 088 938 (96.0%) were male, 3558 (0.3%) were American Indian or Alaska Native, 8845 (0.8%) were Asian or Pacific Islander, 162 934 (14.4%) were Black, 61 264 (5.4%) were Hispanic, and 876 234 (77.3%) were White; the mean (SD) age was 75.9 (8.6) years. Most VMAPs were administered by for-profit insurers (173 [92.0%]; SMD, 0.42), including Aetna (46 [24.9%]), Humana (36 [19.5%]), and United HealthCare (49 [26.5%]). Compared with veterans in other MA plans, veterans in VMAPs were slightly younger (mean [SD] age, 73.7 [8.0] years vs 76.3 [8.7] years; SMD, 0.31), more likely to be Black (34 837 [19.4%] vs 128 097 [13.4%]; SMD, 0.18), and more likely to have zero cost sharing for VHA services (ie, priority group 1) (62 056 [34.6%] vs 195 688 [20.5%]; SMD, 0.40). VMAPs were more likely than other MA plans to offer $0 plan premiums (186 [98.9%] vs 2064 [60.0%]; SMD, 1.10), and Medicare Part B premium reductions (140 [74.5%] vs 298 [8.7%]; SMD, 1.80), averaging $33 more in cash back benefits. Only 1 VMAP offered Medicare Part D coverage compared with most other MA plans (1 [0.5%] vs 3293 [95.7%]; SMD, 6.23). VMAPs were more likely than other MA plans to provide comprehensive dental coverage (179 [95.2%] vs 3006 [87.3%]; SMD, 0.28), hearing aids (184 [97.9%] vs 3012 [87.5%]; SMD, 0.40), eyewear (188 [100%] vs 3620 [94.7%]; SMD, 0.33), over-the-counter drug coverage (179 [95.2%] vs 2831 [82.2%]; SMD, 0.42), and meal benefits (151 [80.3%] vs 2348 [68.2%]; SMD, 0.28).

**Conclusions and Relevance:**

This study found that MA insurers—specifically VMAPs—engaged in targeted marketing to veterans, offering $0 premiums, cash back benefits, and supplemental benefits. However, nearly all VMAPs excluded Medicare Part D, likely designed to attract veteran enrollees who use VHA care, making them low-cost enrollees to the plan. Since the VHA cannot bill plans for Medicare-covered services, VMAPs may be increasing wasteful federal spending.

## Key Points

**Question:**

Are there differences in the plan benefit design, supplemental offerings, and veteran enrollees in veteran Medicare Advantage affinity plans (VMAPs) compared with other Medicare Advantage (MA) plans?

**Findings:**

In this cross-sectional study including more than 1.1 million veterans, compared with 3442 other MA plans, the 188 VMAPs in 2022 were more likely to offer $0 premiums, cash back benefits, over-the-counter drug coverage, and hearing, vision, and dental benefits. However, only 1 VMAP offered Medicare Part D compared with 3293 other MA plans (95.7%).

**Meaning:**

VMAPs use plan design to differentially attract low-cost enrollees, and without Medicare Part D coverage, most VMAP enrollees must rely on the Veterans Health Administration or other coverage sources for prescriptions and related care.

## Teaser

This cross-sectional study examines plan-level differences between Veteran Medicare Advantage affinity plans and other Medicare Advantage plans and characteristics of their veteran enrollees.

## Introduction

Medicare Advantage (MA), the managed care option to traditional fee-for-service Medicare, has been the dominant choice of Medicare coverage among Medicare beneficiaries since 2023. Amidst MA growth, there has been a proliferation of MA plan options, with the average Medicare beneficiary facing 43 MA plans to choose from.^1^ While insurers may strategically design their plans to offer supplemental benefits to differentiate themselves from competitors, in recent years, insurers have engaged in direct marketing to particular subgroups of people by way of MA affinity plans that are designed to appeal to those subgroups.^2,3,4^

Veteran MA affinity plans (VMAPs) are a rapidly emerging type of affinity plan that focus on enrolling veterans. MA insurers have launched plans like Humana Honor in 2020 and United Patriot Plan in 2021 with web-based imaging and marketing materials depicting veterans.^5,6^ Many Medicare-covered veterans are also enrolled in the Veterans Health Administration (VHA) for care, and recent work has raised concern that MA plans are targeting veterans who receive their care through VHA facilities as a way of increasing their profitability.^7,8^ MA plans receive full monthly capitated rates for their veteran enrollees from the US Centers for Medicare & Medicaid Services (CMS), but they do not reimburse the VHA system for any Medicare-covered service that occurs in VHA facilities since the VHA is not allowed to bill Medicare for reimbursement. Therefore, MA plans receive Medicare payments for their veteran enrollees who receive VHA care that would otherwise have been paid for by an MA plan.^9,10,11^

In 2022, 1.3 million VHA enrollees were enrolled in MA, which accounts for more than 1 in 3 veterans who are dual Medicare-VHA enrollees.^12^ As more veterans select MA, the potential for wasteful and unintended, duplicative federal spending will grow, as could MA plans’ profit from CMS overpayments.^8,9,13,14^ Despite these concerns, we have limited evidence characterizing why veterans increasingly choose to enroll in MA plans. While MA plans have engaged in market segmentation with special needs plans for specific groups like Medicare-Medicaid dual-eligible individuals and other populations with chronic conditions, MA plans are also increasingly tailoring their benefit design and supplemental benefits to encourage certain kinds of beneficiaries to enroll.^15,16,17^ Prior work has shown how plans have used fitness memberships to enroll healthier beneficiaries to minimize the risk and costs of care for the plan.^18^ While current marketing strategies suggest that VMAPs are designed to enhance and complement VHA care, the extent to which customization of plan benefit design and supplemental offerings differ in VMAPs vs other MA plans is currently unknown.^5,19^ A comprehensive understanding of potential mechanisms that influence veterans to enroll in VMAPs can help inform VHA and CMS policymakers as they consider health reforms that may improve the care of veterans dually covered by Medicare and the VHA system.

Using national Medicare and VHA data, we sought to answer the following key questions. First, are there significant differences in the plan benefit design and supplemental benefit offerings of VMAPs compared with other MA plans? Second, what are the characteristics of VHA enrollees who enroll in VMAPs vs those in other MA plans?

## Methods

This evaluation was conducted as a quality improvement activity for VHA and was deemed by the US Department of Veterans Affairs Research & Development Committee at the VA Boston Healthcare System not to be human subjects research; therefore, it was not subject to institutional review board review. This work followed the Strengthening the Reporting of Observational Studies in Epidemiology (STROBE) reporting guideline for observational studies.

### Data

Using the publicly available CMS MA Landscape files from 2022, we created a novel directory of VMAPs. First, we identified MA plans with names that included 19 specific words commonly associated with US veterans, like *courage*, *eagle*, and *honor* (eMethods in Supplement 1). Second, we searched for evidence to confirm these plans were directly targeting veterans. We searched insurer websites, marketing materials, news reports, press releases, and other plan coverage documentation for language suggestive of a plan being complementary to one’s VHA benefits or designed for veterans. For any plan in which we could not identify documentation specifically targeting veterans, we deidentified the plan as a VMAP. Plans not identified as VMAPs were designated as other MA plans. We excluded employer plans, cost plans, special needs plans, Program of All Inclusive Care for the Elderly plans, private fee-for-service plans, Medicare Savings Accounts plans, and any plan offered in the US territories because some of these plans have varying rules and regulations for payment that differ from the most common, local coordinated care plans (like health maintenance organizations [HMOs] and preferred provider organizations [PPOs]).

Next, we linked our plan directory data to the Medicare Beneficiary Summary Files and the VA Planning Systems Support Group geocoded enrollee files to identify dually covered enrollees in the VHA system and MA. Beneficiary demographic characteristics (age, sex, reason for Medicare entitlement, and race and ethnicity determined using the Research Triangle Institute Race Code) were identified in the Medicare Beneficiary Summary Files; rurality and veterans’ priority group score were obtained from the VA Planning Systems Support Group geocoded files (eMethods in Supplement 1). Priority groups are VHA eligibility determinations primarily based on enrollees’ service-connected disabilities and income.^20^ Veterans assigned the highest priority (ie, priority group 1) do not incur any copayment for VHA care, whereas veterans assigned lower priority groups (ie, priority groups 2 to 8) incur some copayments for non–service-connected conditions (eMethods in Supplement 1).

Finally, we used CMS’ public landscape, plan directory, and plan benefit package (PBP) files to identify the plan-level characteristics and supplemental benefit offerings.^21^ Plan-level characteristics from the landscape and directory files included plan type, parent contract organization, contracts’ tax status, enrollment numbers, average monthly plan premiums (for Medicare Part C and/or Medicare Part D), whether the plan offers $0 monthly premiums, CMS star ratings that are meant to reflect the underlying quality of a contract, and prescription drug benefits. Supplemental benefits from the plan benefit package data included Medicare Part B premium reductions, vision, dental, hearing, and other preventive/enhanced services, like acupuncture, over-the-counter drugs, meals, and fitness benefits. Further detail on the sample selection can be found in the eFigure in Supplement 1.

### Statistical Analysis

We first identified the number of VMAPs vs other MA plans in 2022 and the number of VHA enrollees in each group. We then compared plan-level characteristics, supplemental benefit offerings, and veteran enrollee characteristics of VMAPs vs other MA plans using standardized mean differences (SMDs), where SMDs less than 0.1 were considered to be negligible differences.^22^ Data were collected and analyzed between April 2023 to August 2024 using Stata version 18.0 (StataCorp) and SAS Enterprise Guide version 8.3 (SAS Institute).

## Results

The sample included 188 VMAPs with 179 449 veteran enrollees and 3442 other MA plans with 954 581 veteran enrollees. A total of 1 088 938 (96.0%) were male, 3558 (0.3%) were American Indian or Alaska Native, 8845 (0.8%) were Asian or Pacific Islander, 162 934 (14.4%) were Black, 61 264 (5.4%) were Hispanic, and 876 234 (77.3%) were White; the mean (SD) age was 75.9 (8.6) years. Of the 188 VMAP plans, 90 (47.9%) included the term *patriot*, 46 (24.5%) included the term *eagle*, and 36 (19.1%) included the term *honor*. The remaining 16 VMAPs (8.5%) used the terms *courage*, *freedom*, *liberty*, *salute*, *valiance*, *valor*, or *veteran* in their plan name (Table 1). Last, we observed lower average plan enrollment in VMAPs relative to other MA plans by approximately 3270 enrollees (mean [SD] 1596 [2664.0] vs 4866 [10 027.0]; SMD, 0.44) (Table 2).

**Table 1.** Review of Key Terms in Plan Names of Veteran Medicare Advantage Affinity Plans (VMAPs) Marketing to Veterans in 2022

Marketing term	VMAP frequency, No. (%)
Courage	1 (0.5)
Eagle	46 (24.5)
Freedom	1 (0.5)
Honor	36 (19.1)
Liberty	2 (1.1)
Patriot	90 (47.9)
Salute	3 (1.6)
Valiance	6 (3.2)
Valor	2 (1.1)
Veteran	1 (0.5)
Total	188 (100)

**Table 2.** Plan-Level Characteristics of Veteran Medicare Advantage Affinity Plans (VMAPs) vs Other Medicare Advantage (MA) Plans in 2022^a^

Characteristic	Other MA plans (n = 3442)	VMAPs (n = 188)	SMD
Organization detail			
Parent contract			
Aetna	464 (13.5)	46 (24.9)	0.93
Humana	442 (12.8)	36 (19.5)	
United HealthCare	436 (12.7)	49 (26.5)	
WellCare	311 (9.0)	33 (17.8)	
Other	1789 (52)	24 (11.4)	
Tax status			
For-profit	2655 (77.1)	173 (92.0)	0.42
Non-profit	787 (22.9)	15 (8.0)	
Plan design and quality			
Plan type			
PPO	1294 (37.6)	112 (59.6)	0.45
HMO	2148 (62.4)	76 (40.4)	
Medicare Part D prescription drug benefit coverage			
None	149 (4.3)	187 (99.5)	6.23
Basic	65 (1.9)	0	
Enhanced	3228 (93.7)	1 (0.5)	
CMS contract star rating, mean (SD)	4.16 (0.55)	4.10 (0.50)	0.13
Plan cost sharing			
Zero $ premium plan			
No	1378 (40.0)	2 (1.1)	1.10
Yes	2064 (60.0)	186 (98.9)	
Monthly plan premium, mean (SD), $	24.50 (44.50)	0.40 (3.93)	0.76
Use of plan rebate toward Medicare Part B premium reduction			
No	3144 (91.3)	48 (25.5)	1.80
Yes	298 (8.7)	140 (74.5)	
Medicare Part B premium reduction amount, mean (SD), $	5.80 (21.66)	38.96 (32.18)	1.21
Maximum out-of-pocket amount, mean (SD), $	5165.00 (1701.60)	5392.00 (1229.40)	0.15
Monthly enrollment, mean (SD)	4866 (10 027)	1596 (2664)	0.44

Abbreviations: CMS, US Centers for Medicare & Medicaid Services; HMO, health maintenance organization; PPO, preferred provider organization; SMD, standardized mean difference.

^a^ Employer, cost, special needs plans, Medicare Savings Account plans, and plans in US territories were excluded from the analysis.

### Plan-Level Characteristics

VMAPs and other MA plans had meaningful differences between their contracting organizations, benefit design, and cost sharing. Compared with other MA plans, VMAPs were most likely to be administered by for-profit insurers (173 [92.0%] vs 2655 [77.1%]; SMD, 0.42). Compared with other MA plans, the main insurance companies offering VMAPs included Aetna (46 [24.9%] vs 464 [13.5%]; SMD, 0.93), Humana (36 [19.5%] vs 442 [12.8%]; SMD, 0.93), United HealthCare (49 [26.5%] vs 436 [12.7%]; SMD, 0.93), and WellCare (33 [17.8%] vs 311 [9.0%]; SMD, 0.93). VMAPs were also more likely to be PPOs (112 [59.6%] vs 1294 [37.6%]), whereas more than half of the other MA plans were HMOs (76 [40.4%] vs 2148 [62.4%]) (SMD, 0.45). Notably, only 1 VMAP (0.5%) compared with nearly all other MA plans (3293 [95.7%]) offered Medicare Part D coverage (SMD, 6.23). Additionally, VMAPs’ contract-level star ratings were slightly lower on average than other MA plans’ star ratings (mean [SD] rating, 4.10 [0.50] stars vs 4.16 [0.55] stars; SMD, 0.13) (Table 2).

VMAP and other MA plans exhibited pronounced differences in cost sharing structure. Nearly all VMAPs (186 [98.9%]) offered $0 plan premiums for their enrollees compared with 2064 other MA plans (60.0%; SMD, 1.10). VMAPs were also more likely to offer Medicare Part B premium reductions (140 [74.5%] vs 298 [8.7%]; SMD, 1.80), with a mean (SD) of $38.96 ($32.18) in cash back benefits vs $5.80 ($21.66) in other MA plans (SMD, 1.21). VMAPs also had slightly higher mean (SD) maximum out-of-pocket-cost limits ($5392.00 [$1229.40] vs $5165.00 [$1701.60]; SMD, 0.15) (Table 2).

### Differences in Supplemental Benefit Offerings

VMAPs were more likely to offer certain supplemental benefits than other MA plans (Table 3). VMAPs were more likely to offer comprehensive dental coverage (179 [95.2%] vs 3006 [87.3%]; SMD, 0.28) and preventive dental coverage (185 [98.4%] vs 3267 [94.9%]; SMD, 0.20). VMAPs were also more likely to offer hearing examinations (185 [98.4%] vs 3120 [90.6%]; SMD, 0.35) and hearing aids (184 [97.9%] vs 3012 [87.5%]; SMD, 0.40) relative to other MA plans. Additionally, all VMAPs offered eyewear and eye examination services compared with 3620 (94.7%; SMD, 0.33) and 3401 (98.8%; SMD, 0.16) other MA plans, respectively. Meal benefits were also a service more likely covered in VMAPs than in other MA plans (151 [80.3%] vs 2348 [68.2%]; SMD, 0.28). Although Medicare Part D drug benefits were not as available in VMAPs, the largest meaningful difference in benefits between VMAPs and other MA plans was coverage for over-the-counter medications (179 [95.2%] vs 2831 [82.2%]; SMD, 0.42) (Table 3). No meaningful differences were detected in the rates of offering fitness and gym memberships, acupuncture benefits, and other enhanced preventive services (Table 3).

**Table 3.** Supplemental Benefit Offerings of Veteran Medicare Advantage Affinity Plans (VMAPs) vs Other Medicare Advantage (MA) Plans in 2022^a^

Benefit	Other MA plans (n = 3442)	VMAPs (n = 188)	SMD
Acupuncture services	1223 (35.5)	62 (33.0)	0.05
Fitness benefits	1792 (52.1)	98 (52.1)	0
Enhanced preventive services	3434 (99.8)	188 (100)	0.06
Meal services	2348 (68.2)	151 (80.3)	0.28
Over-the-counter medications	2831 (82.2)	179 (95.2)	0.42
Eyewear	3620 (94.7)	188 (100)	0.33
Eye examinations	3401 (98.8)	188 (100)	0.16
Hearing aids	3012 (87.5)	184 (97.9)	0.40
Hearing examinations	3120 (90.6)	185 (98.4)	0.35
Comprehensive dental services	3006 (87.3)	179 (95.2)	0.28
Preventive dental services	3267 (94.9)	185 (98.4)	0.20

Abbreviation: SMD, standardized mean difference.

^a^ Employer, cost, special needs plans, Medicare Savings Account plans, and plans in US territories were excluded from the analysis.

### Veteran Enrollee Characteristics

Although VMAPs comprise just 5% of the total MA plans in our sample and exhibited a smaller share of enrollment, in 2022, 179 449 VHA enrollees were enrolled in VMAPs corresponding to nearly 60% of total VMAP enrollees. In contrast, the 954 581 VHA enrollees enrolled in other MA plans only accounted for 5.7% of total enrollees in other MA plans in 2022 (Figure).

**Figure.**
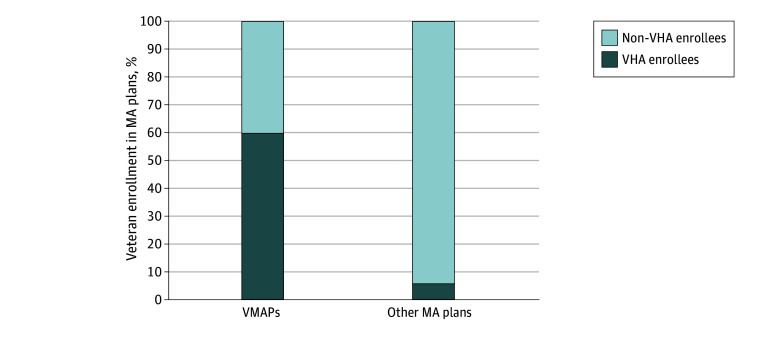
Veterans Health Administration (VHA) Veteran Enrollment in Veteran Medicare Advantage Affinity Plans (VMAPs) and Other Medicare Advantage (MA) Plans in 2022 The proportion of VHA-enrolled veterans and non–VHA enrollees in VMAPs and other MA plans. VHA-enrolled veterans included dually enrolled beneficiaries that could be identified between the Medicare Beneficiary Summary Files and Veterans Affairs Planning Systems Support Group files, excluding those who were ineligible for VHA benefits (priority groups 8e and 8g). Non–VHA enrollees could only be identified in Medicare Beneficiary Summary Files.

VMAP-enrolled veterans differed from veterans enrolled in other MA plans across all demographic categories except sex. VMAP-enrolled veterans were slightly younger than other MA plan–enrolled veterans (mean [SD] age, 73.7 [8.0] years vs 76.3 [8.7] years; SMD, 0.31). A larger proportion of VMAP-enrolled veterans were Black (34 837 [19.4%] vs 128 097 [13.4%]; SMD, 0.18), disabled (53 706 [29.9%] vs 216 166 [22.6%]; SMD, 0.17), and assigned to VHA priority group 1 (62 056 [34.6%] vs 195 688 [20.5%]; SMD, 0.40) compared with veterans enrolled in other MA plans. Veterans in VMAPs were also more likely to live in rural census tracts (68 468 [38.2%] vs 270 293 [28.3%]; SMD, 0.25) (Table 4).

**Table 4.** Characteristics of Veterans Health Administration (VHA) Enrollees in Veteran Medicare Advantage Affinity Plans (VMAPs) vs Other Medicare Plans (MA) Plans in 2022^a^^,^^b^

Characteristic^c^^,^^d^	Veteran enrollees in other MA plans (n = 954 581)	Veteran enrollees in VMAPs (n = 179 449)	SMD
Age, mean (SD), y	76.3 (8.7)	73.7 (8.0)	0.31
Sex			
Female	35 793 (3.7)	9299 (5.2)	0.07
Male	918 788 (96.3)	170 150 (94.8)	
Race and ethnicity^e^			
American Indian or Alaska Native	2531 (0.3)	1027 (0.6)	0.18
Asian or Pacific Islander	7648 (0.8)	1197 (0.7)	
Black	128 097 (13.4)	34 837 (19.4)	
Hispanic	53 550 (5.6)	7714 (4.3)	
Non-Hispanic White	744 701 (78.0)	131 533 (73.3)	
Other race	5192 (0.5)	935 (0.5)	
Unknown race	12 862 (1.3)	2206 (1.2)	
Original reason for Medicare entitlement			
Age	736 839 (77.2)	125 289 (69.8)	0.17
Disability	216 166 (22.6)	53 706 (29.9)	
End-stage kidney disease	810 (0.1)	219 (0.1)	
Disability and end-stage kidney disease	766 (0.1)	235 (0.1)	
Priority group			
1	195 688 (20.5)	62 056 (34.6)	0.40
2	52 873 (5.5)	12 078 (6.7)	
3	131 415 (13.8)	25 708 (14.3)	
4	26 803 (2.8)	5298 (3.0)	
5	199 836 (20.9)	35 461 (19.8)	
6	62 770 (6.6)	6890 (3.8)	
7	74 531 (7.8)	7080 (3.9)	
8	210 665 (22.1)	24 878 (13.9)	
Rurality			
Urban	653 480 (68.5)	101 644 (56.6)	0.25
Rural	270 293 (28.3)	68 468 (38.2)	
Highly rural	30 061 (3.2)	9237 (5.2)	
Island	40 (<0.1)	10 (<0.1)	

Abbreviation: SMD, standardized mean difference.

^a^ Employer, cost, special needs plans, Medicare Savings Account plans, and plans in US territories were excluded from the analysis.

^b^ VHA-enrolled veterans included dually enrolled beneficiaries that could be identified between the Medicare Beneficiary Summary Files and Veterans Affairs Planning Systems Support Group files, Veterans ineligible for VHA benefits (priority groups 8e and 8g) were excluded from the analysis.

^c^ Age, sex, race and ethnicity, and original reason for Medicare entitlement variables were obtained from the 100% Medicare Beneficiary Summary File from 2022; rurality and priority group status were obtained from the Veterans Affairs Planning Systems Support Group files.

^d^ See the eMethods in Supplement 1 for additional detail on race and ethnicity, priority group, and rurality variables.

^e^ Race and ethnicity were determined using the Research Triangle Institute Race Code.

## Discussion

In this national analysis of MA plans, we observed that MA insurers used military-associated terms in their plan names and marketing materials to target US veterans. Nearly all the insurers offering VMAPs were for-profit entities, including companies like Aetna, Humana, and United HealthCare. These VMAPs were more likely to offer $0 premiums, cash back benefits, and additional supplemental benefits than other MA plans. Additionally, nearly 60% of enrollees in VMAPs were veterans dually covered in the VHA system, and more than one-third of these dually covered veterans belonged to priority group 1, which means they faced no cost sharing when receiving care in the VHA. As long as insurers capture veterans’ diagnoses for capitation payment and risk adjustment purposes, plans could financially benefit if their enrollee used VHA care, effectively making them a low-cost enrollee to the plan.^23^ These results are highly suggestive that MA plans are using plan benefit design and supplemental offerings to target veterans who are more likely to rely on the VHA for their care.

The most prominent finding that insurers are customizing their plan benefit design to attract low-cost enrollees is the exclusion of Medicare Part D drug coverage in all but 1 VMAP. By excluding Medicare Part D coverage from their benefits, VMAPs attract beneficiaries who can receive their prescriptions from another source, like the VHA. To use VHA pharmacies, veterans require some VHA contact for their care and filling of medications.^24,25^ This increases the likelihood that veterans enrolled in a VMAP are receiving VHA care for which the VMAP does not pay. In contrast, more than 95% of the other MA plans offered Medicare Part D drug coverage. The composition of VHA enrollees in VMAPs provides additional evidence that plans are enrolling veterans more likely to use the VHA for care, as a large proportion of the VMAP enrollees belonged to VHA priority group 1. Priority group 1 enrollees are often more reliant on the VHA than other priority groups for their health care needs, meaning the federal government may be more likely to incur wasteful spending for this priority group if dually enrolled in MA plans.^9,20,26^ Our findings suggest that MA insurers are operating in unique ways to attract and enroll veterans into VMAPs, atypical from other forms of selection that have used benefits like gym memberships to attract healthier and less costly beneficiaries.^18^

VMAPs were more likely than other MA plans to provide more generous supplemental benefits and cost sharing. One motivation might be to attract enrollees into their plans, although most of these supplemental benefits, including vision and hearing coverage, are accessible in the VHA by at least a subset of veterans, like those in priority group 1.^27^ Eligibility for VHA dental coverage, however, differs from other VHA services, and not all VHA enrollees qualify for VHA-covered dental care.^28^ However, veterans may seek supplemental benefits outside of the VHA to expand choice. It is unknown whether veterans receive these benefits in the VHA or VMAP, but it is possible that the plan name and marketing of VMAPs may be sufficient in getting veterans to enroll. The sizable Medicare Part B premium reductions may also pose an additional incentive for enrollment. Relative to other MA plans, VMAPs offered an additional $38 per month on average through the Medicare Part B premium reduction, which can be applied either to one’s monthly social security check or monthly Medicare Part B premium payment. Annually, this adds up to an extra $450 in savings or cash back that veterans could get just by choosing a VMAP over another MA plan.

Further work is needed to understand the benefit utilization, clinical outcomes, and quality of VMAPs, and there may be important equity issues to explore. In our evaluation, we found a larger proportion of Black veterans, veterans with disabilities, and veterans living in rural areas enrolling in VMAPs relative to other MA plans. While dual VMAP and VHA coverage may present opportunities for enhanced, comprehensive care and access to services, seeking care in 2 systems may lead to poorly coordinated, fragmented care.^29^ Consequently, it is important to evaluate whether VMAPs’ tailored benefits yield improved outcomes and accessibility compared with other MA plans using encounter data. Similarly, it is worth examining whether veterans are receiving and using the supplemental benefits offered within VMAPs, by the VMAP and/or by VHA. There is substantial concern that many beneficiaries do not actually receive purported supplemental benefits, and the use of prior authorizations for these services may be a barrier.^30^

This work contributes to the growing body of literature on veteran enrollment in MA plans in several ways. Our evaluation is unique in that it focuses on the identification of MA plans specifically marketing themselves to veterans, presenting a more salient and patient-centered approach to what veterans may see when making their plan choice. These findings have important implications for CMS and VHA leadership interested in improving the care for dually covered veterans in MA. High enrollment of veterans with VHA benefits has the potential to result in unintentional duplicative payments that waste taxpayer resources.^7,8,9^ Although it is beyond the scope of this work to quantify the scale of duplicative spending in VMAPs, recent work is beginning to do so. Ma et al^8^ found that about 1 in 5 veterans enrolled in MA plans with high veteran enrollment (at least 20% of their plan enrollees being veterans) did not submit a single Medicare claim that was paid by the plan. The study estimated that CMS paid more than $1.32 billion to these MA plans for providing no Medicare services for the group of veteran enrollees in 2020. Other work by Meyers et al^7^ identified $78 billion in VHA spending for dual MA enrollees between 2011 and 2020. Therefore, if VMAPs are strategically designed to attract veterans who use VHA care, the scale of wasteful and unintended, duplicate spending may become more prominent as VMAPs grow.

Although VMAPs present customized benefits designed to lower veterans’ Medicare cost sharing and enhance options for care outside of the VHA, additional regulation and monitoring of VMAPs may be warranted to minimize the risk of duplicate payments and safeguard taxpayer resources. CMS may want to ensure that plan payments reflect some expected enrollee use of Medicare services and adjust payments accordingly. To improve the care management and financing of MA and VHA dual enrollees, policymakers may want to consider implementing capitation payment rate adjustments to VMAPs or transitioning VMAPs to a Medicare supplement plan as opposed to a full MA option for dual coverage.^19^

### Limitations

This analysis has some important limitations. Although our approach for identifying VMAPs in the MA marketplace is specific in its primary search of veteran and military-related plan names, this may fail to capture other ways MA plans may be marketing to the veteran population, including the use of insurance agents, brokers, and veteran service organizations like the American Legion. Similarly, the timing of our VMAP directory construction may have limited the number of plans we could identify as advertising to veterans during our study period. If a plan had a military-related term in their plan name but lacked online documentation to validate its marketing to veterans at the time of our search, that plan was excluded from our analysis. We also could not tell whether plans exclusively targeted VHA enrollees or the veteran population more generally, including TRICARE veterans in the Military Health System and other veterans not enrolled in VHA benefits. We lacked the data to identify non-VHA veterans and their characteristics and trends in enrollment. Last, this work did not assess the impact of VMAPs on utilization or care outcomes for enrollees compared with other MA plans.

## Conclusions

In this study, MA insurers offered VMAPs that engaged in targeted marketing and unique plan benefit customizations—including foregoing Medicare Part D coverage but offering more generous Medicare Part B premium reductions and supplemental benefits—to attract veterans who are more likely to rely on the VHA system for their health care needs. Ongoing enrollment growth among veterans into VMAPs may increase wasteful and unintended, duplicative federal spending while increasing MA plan profits.

## Notes

Supplement 1. **eMethods.** Datasets and Variables **eFigure.** Sample Selection Flowchart

Supplement 2. Data Sharing Statement
