# Supplementary material for: Characteristics and Benefit Design of Veteran Medicare Advantage Affinity Plans
Source: JAMA Health Forum. 2025 Mar 28;6(3):e250159. doi: 10.1001/jamahealthforum.2025.0159 (PMC11953753; doi:10.1001/jamahealthforum.2025.0159)
Supplement: Supplement 1. — eMethods. Datasets and Variables eFigure. Sample Selection Flowchart [file jamahealthforum-e250159-s001.pdf]

Supplemental Online Content

Dorneo A, Ma Y, Garrido MM, et al. Characteristics and benefit design of veteran Medicare Advantage affinity plans. *JAMA Health Forum.* 2025;6(3):e250159. doi:10.1001/jamahealthforum.2025.0159

eMethods. Datasets and Variables

eFigure. Sample Selection Flowchart

This supplementary material has been provided by the authors to give readers additional information about their work.

© 2025 Dorneo A et al. *JAMA Health Forum*.

eMethods. Datasets and Variables

Key terms searched to identify Veteran Medicare Advantage Affinity Plans (VMAPs) in plan name

To identify our VMAPs, we searched a total of 19 terms that we found to be synonymous and associated with veterans in the MA Landscape files. These terms included: Army, Brave, Courage, Eagle, Freedom, Hero, Honor, Liberty, Military, Patriot, Proud, Salute, Shield, Tribute, Valiance, Valor, Vet, Veteran, and Warrior. Of these terms, only 10 of them returned plans that we could validate against existing online documentation that they were marketed to the veteran population, as described in the Methods section and Table 1.

Although we identified several other plans in our directory with the terms "Freedom", "Liberty", and "Shield" in their plan name, we could not determine whether these were specifically marketed to the veteran population for the year of our analysis. For example, "Freedom" may be associated with a broader term used to attract Medicare beneficiaries, signaling a "freedom" to see various providers or having "freedom" to access various specialties. Similarly, most of the "Shield" plans identified in plan name were associated with the Blue Cross Blue Shield organization.

The Research Triangle Institute (RTI) Race and Ethnicity Code

The race and ethnicity variable obtained from the Medicare Beneficiary Summary File (MBSF) was developed by RTI International. This variable is constructed using an algorithm that predicts race and ethnicity based on beneficiary's first and last name from their Social Security information. This approach enhances the Social Security race and ethnicity classification and is able to identify more beneficiaries as either Hispanic or Asian. The RTI race/ethnicity categories include Unknown, Non-Hispanic White, Black (or African-American), Other, Asian/Pacific Islander, Hispanic, and American Indian/ Alaska Native. Beneficiaries in the "Other" category included those who could not be classified as Non-Hispanic White, Black, Asian/Pacific Islander, Hispanic, or American Indian/Alaska Native. Race and ethnicity variables were included to identify whether there were differences or inequities among veterans enrolling in VMAPs vs. other MA plans.

Source: Research Data Assistance Center (ResDAC). Research Triangle Institute (RTI) Race Code. https://resdac.org/cms-data/variables/research-triangle-institute-rti-race code#:~:text=Beneficiary%20race%20code%20(modified%20using,Hispanic%20or%20Asian%20in%20 origin. (2024)

VHA Priority Group Designation

Of the eight enrollment priority groups obtained from the Veteran PSSG Enrollee file, veterans in priority group 1 have the highest enrollment priority and priority groups 7-8 have the lowest enrollment priority. Veterans eligible for **priority group 1** have VHA-rated service-connected disabilities that are considered 50% or more disabling, impacting their ability to work. **Priority groups 2-3** are also based on service-connected disabilities that are rated 30% or 40% and 10% or 20% disabling, respectively. Veterans may also be eligible for priority group 3 if they are a former Prisoner of War (POW) or have received a Purple Heart Medal, the Medal of Honor, or other similar awards. **Priority group 4** veterans are typically homebound and require some VA aid and attendance. Veterans in **priority group 5** often have lower incomes that also qualify them for Medicaid-eligibility, and veterans in **priority group 6** apply to those who have served in Vietnam, the Persian Gulf War, or have been exposed to radiation and other toxic chemicals during their active service. Lastly, veterans in **priority group 7-8** typically have higher-than-VHA-average household incomes and pay co-pays for care.

© 2025 Dorneo A et al. *JAMA Health Forum*.

Source: US Department of Veterans Affairs. https://www.va.gov/health-care/eligibility/priority-groups/ (2024)

VHA Urban, Rural Classification

Obtained from the Veteran PSSG Enrollee file, the VHA's urban/rural classification is based on the census tract in which the veteran resides. The VA's Office of Rural Health (ORH) uses the Rural-Urban Commuting Area codes (RUCA) framework and scores to classify whether a veteran is considered to live in an urban, rural, highly rural area, or on an insular island. Veterans in census tracts with a RUCA score of 1.0 or 1.1 are determined to be living in urban areas. Veterans in census tracts with RUCA score of 10 are considered to live in a highly rural area, and veterans in census tracts with scores between 1.1 and 10 are considered rural. Though RUCAs are not assigned for insular islands like US territories, the ORH is able to separately identify these designations.

Source: US Department of Veterans Affairs. Office of Rural Health (ORH) Fact Sheet: How to Define Rurality. https://www.ruralhealth.va.gov/docs/ORH_RuralityFactSheet_508.pdf (2024)

© 2025 Dorneo A et al. *JAMA Health Forum*.

eFigure. Sample Selection Flowchart

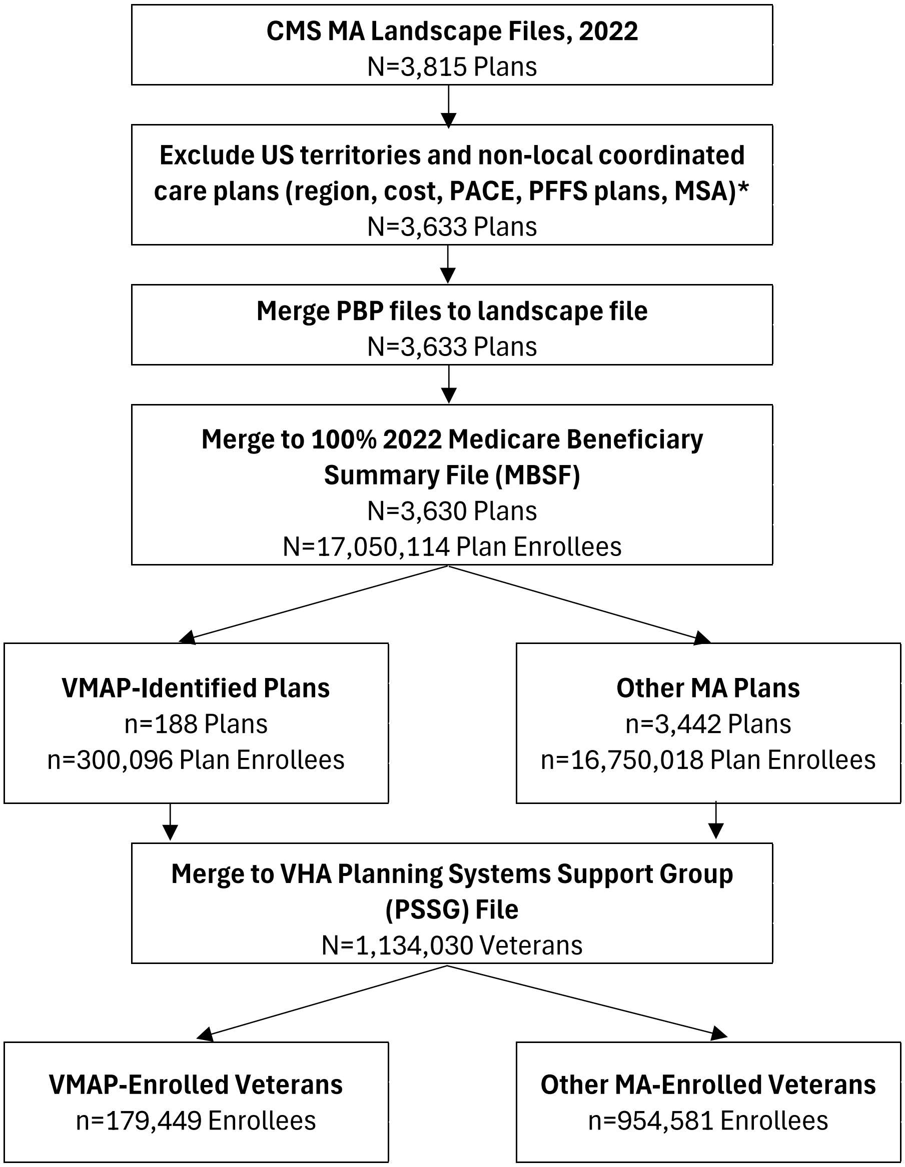

Notes: From the CMS landscape file, we aggregated observations at the contract-plan level.

© 2025 Dorneo A et al. *JAMA Health Forum*.
