# Supplementary material for: Characteristics and Benefit Design of Veteran Medicare Advantage Affinity Plans
Source: JAMA Health Forum. 2025 Mar 28;6(3):e250159. doi: 10.1001/jamahealthforum.2025.0159 (PMC11953753; doi:10.1001/jamahealthforum.2025.0159)
Supplement: Supplement 2. — Data Sharing Statement [file jamahealthforum-e250159-s002.pdf]

Data Sharing Statement

Dorneo. Characteristics and Benefit Design of Veteran Medicare Advantage Affinity Plans. *JAMA Health Forum*. Published March 28, 2025. doi:10.1001/jamahealthforum.2025.0159

Data
Data available: No

Additional Information
Explanation for why data not available: The list of plans used in the analysis can be made available upon request via email to the corresponding author.
